# Chondrogenic Differentiation of Pluripotent Stem Cells under Controllable Serum-Free Conditions

**DOI:** 10.3390/ijms20112711

**Published:** 2019-06-02

**Authors:** Michał Stefan Lach, Joanna Wroblewska, Katarzyna Kulcenty, Magdalena Richter, Tomasz Trzeciak, Wiktoria Maria Suchorska

**Affiliations:** 1 Radiobiology Lab, The Greater Poland Cancer Centre, Garbary 15 Street, 61-866 Poznan, Poland; katarzyna.kulcenty@wco.pl (K.K.); wiktoria.suchorska@wco.pl (W.M.S.); 2 The Postgraduate School of Molecular Medicine, Medical University of Warsaw, Księcia Trojdena 2a Street, 02-109 Warsaw, Poland; 3 Department of Electroradiology, Poznan University of Medical Sciences, Garbary 15 Street, 61-866 Poznan, Poland; 4 Department of Pathology, Poznan University of Medical Sciences, The Greater Poland Cancer Centre, Garbary 15 Street, 61-866 Poznan, Poland; joanna.wroblewska@wco.pl; 5 Department of Orthopedics and Traumatology, Poznan University of Medical Sciences, 28 Czerwca 1956r 135/147 Street, 61-544 Poznan, Poland; mrichter@ump.edu.pl (M.R.); doktortrzeciak@gmail.com (T.T.)

**Keywords:** articular cartilage, pluripotent stem cells, embryoid bodies, chondrogenic differentiation

## Abstract

The repair of damaged articular cartilage using currently available implantation techniques is not sufficient for the full recovery of patients. Pluripotent stem cells (iPSC)-based therapies could bring new perspectives in the treatment of joint diseases. A number of protocols of in vitro differentiation of iPSC in chondrocytes for regenerative purposes have been recently described. However, in order to use these cells in clinics, the elimination of animal serum and feeder cells is essential. In our study, a strictly defined and controllable protocol was designed for the differentiation of pluripotent stem cells (BG01V, ND 41658*H, GPCCi001-A) in chondrocyte-like cells in serum- and a feeder cell-free system, using the embryoid bodies step. The extension of the protocol and culture conditions (monolayer versus 3D culture) was also tested after the initial 21 days of chondrogenic differentiation. Promotion of the chondrogenic differentiation in 3D culture via the elevated expression of genes related to chondrogenesis was achieved. Using immunofluorescence and immunohistochemistry staining techniques, the increased deposition of the specific extracellular matrix was indicated. As a result, chondrocyte-like cells in the early stages of their differentiation using pellet culture under fully controlled and defined conditions were obtained.

## 1. Introduction

Physical damage or age-related degenerative diseases of articular cartilage repair remains challenging for physicians [1]. This is related to specific tissue structure, where the lack of vascularization, innervation, and low cellular density reduce its self-healing capacity. Additionally, articular cartilage consists of dense fibrous architecture creating a functional extracellular matrix (ECM), which maintains their biomechanical and specific microenvironment properties [2,3,4]. Existing therapeutic approaches, such as microfracture or autologous chondrocyte implantation (ACI) are neither able to completely restore large cartilage defects with naïve biomechanical properties, nor to provide long-term improvement [5,6,7]. In consequence, an invasive and expensive procedure of total joint replacement is applied which severely limits patients’ quality of life and requires a complicated revision surgery [8,9]. 

The use of cells such as mesenchymal stromal cells (MSC) and pluripotent stem cells (PSC) introduces new players to the field of regenerative medicine. MSC could be derived from distinct tissues such as bone marrow, fat tissue or cord blood. However, the amount of MSC in these tissues is relatively low and requires further in vitro expansion. This results in increased cell senescence and decrease of their regenerative potential [10,11]. Additionally, the use of MSC in older patients is limited mostly due to their low regeneration capacity and decreased differentiation potential in their chondrogenic lineage. Moreover, the population of MSC decreases in an age-related manner [12]. To overcome these limitations, PSC could be used for regenerative purposes as an alternative. The application of human embryonic stem cells (hESC) raises ethical and controversial issues due to the necessity of destroying the blastocyst to harvest inner cell mass for lineage derivation. Thus, the differentiation of induced pluripotent stem cells (iPSC) seems to be an optimal source of cells. These patient-derived cells not only enable decreasing the risk of rejection of the obtained tissue but also are much less controversial than hESC. However, their stability and safety are still questionable, because there is a lack of information on the long-term effects of their use [13]. Moreover, recent data indicate important differences between iPSC and hESC because of the presence of mutations and epigenetic changes, which creates the necessity to test their safety before application in the clinic [14,15,16,17]. Recent findings have indicated that iPSC heterogeneity is related to donor mutations, the epigenetic memory of reprogrammed cells as well as the system used for iPSC cells production [16,17,18]. Another problem comes from feeder-dependent culture. Due to the use of feeder cells, the variability among PSC clones and batches is high. For this reason, for clinical application, the feeder and xeno-free system should be used for PSC-based therapies. Additionally, the iPSC cells derived under feeder-free conditions are characterized by lower genetic variability and high similarity of gene expression profile to embryonic stem cells [19].

Regardless of these reports, iPSC remains a highly attractive material to design disease models and analyze the steps of differentiation in the desired types of cells. Such an approach could explain the underlying mechanisms of many degenerative processes and enrich our knowledge of developmental biology [20,21,22,23]. For that purpose, reproducible and standardized protocols of iPSC differentiation need to be developed. One of the common issues of tissue engineering based on PSC is the use of serum-based protocols and the presence of feeder cells. This also concerns chondrogenic differentiation protocols from PSC, where at some point of culture fetal bovine serum (FBS) is used [24]. Fetal bovine serum (FBS), with variable batch-to-batch composition, concentration of growth factors, and other bioactive molecules could influence the efficiency of differentiation, and result in the distinct quality of the obtained tissue [25,26]. In the case of animal-derived serum and feeder cells, an increased risk to deliver animal-borne infections remains a crucial issue [26]. Other problems concern the ethical side, especially the derivation process [27]. The aforementioned issues are major factors that disqualify products derived under such conditions from clinical application. 

The aim of this study was to develop a standardized protocol of two-step chondrogenic differentiation using pluripotent stem cell lines; both hESC and iPSC cultured under controllable and serum- and feeder-free conditions.

## 2. Results

### 2.1. The Adaptation of Pluripotent Cell Lines to Serum- and Feeder-Free Conditions

**Figure 1:**
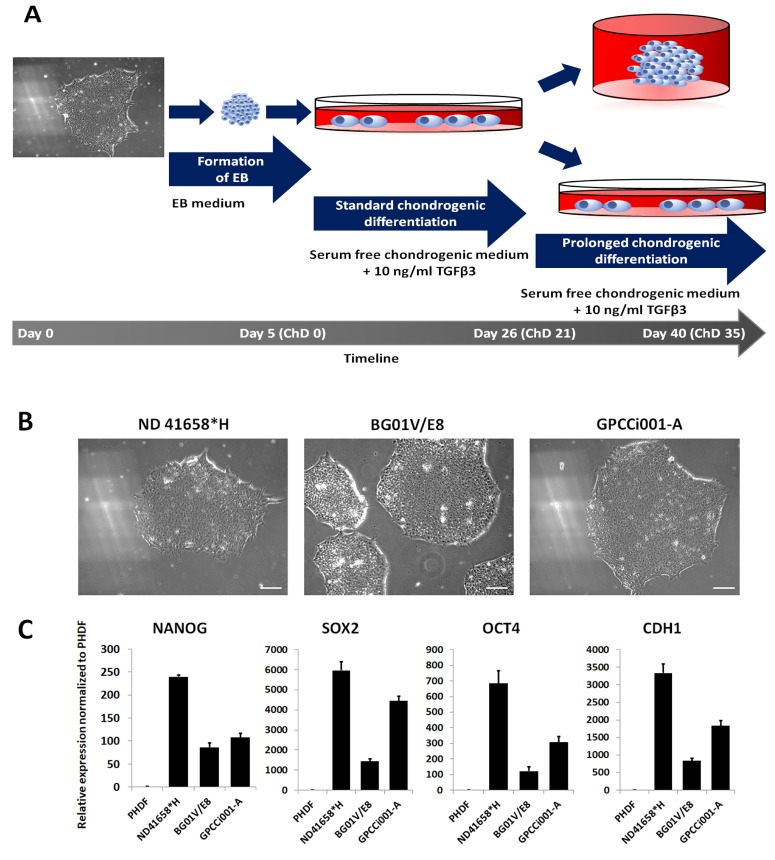
The scheme of the experiment and adaptation of pluripotent cell lines in serum- and feeder-free conditions. (**A**) Pluripotent stem cells (colony of cells, magnification 100×) undergo formation of EBs for five days in suspension culture. The formed EBs were then transferred onto Matrigel™ coated plates for the standard 3-week differentiation in serum-free conditions with supplementation of TGFβ3. The prolonged time of differentiation was used to establish an influence on chondrogenesis. Additionally, 3D culture was performed to compare its effect on differentiation and maturation of chondrogenic like-cells. (**B**) The morphology of pluripotent stem cells adapted to serum- and feeder-free conditions shows that the colonies formed from compacted cells with a high rate of the nucleus to cytoplasm ratio. The white scale bar represents 50 µm. (**C**) The expression of pluripotency markers in adapted pluripotent cell lines indicates their increased expression in comparison with PHDF.

In order to establish a serum-free, reproducible, and low-cost protocol for chondrocyte derivation regardless the origin of PSC lines, two commercially available BGV01 hESC line and ND 41658*H iPS cell line, and GPCCi001-A iPS cell line derived in our laboratory were used [28]. While selected iPS cell lines are cultured in feeder- and serum-free conditions, we adapted BGV01 to those conditions as previously described [28]. The obtained cell line formed compact cell colonies with clear boundaries and a high nucleus to cytoplasm ratio, similar to iPSC cell lines cultured in those conditions (Figure 1B,C). RT-qPCR confirmed the high expression of pluripotent markers (*NANOG, SOX2, OCT4, CDH1*) in comparison with control human primary dermal fibroblasts. 

### 2.2. RT-qPCR Analysis of Differentiated Cells Revealed Early Chondrogenesis

**Figure 2:**
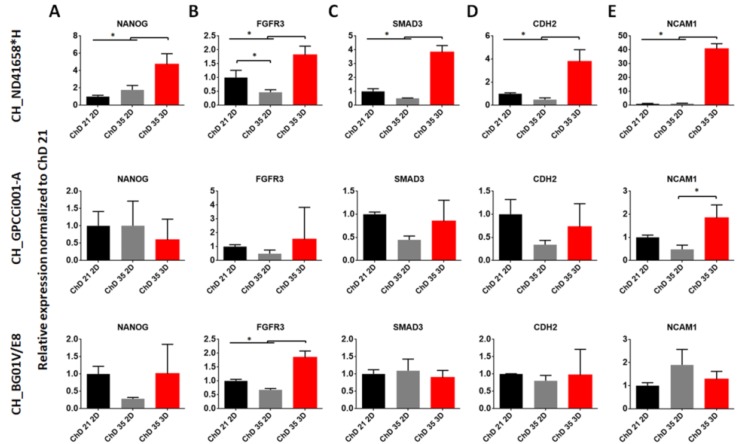
The effect of prolonged and 3D culture on expression of pluripotent (**A**), chondrogenic signaling pathways (**B**,**C**) and mesenchymal condensation (**D**,**E**) marker genes. RT-qPCR represents gene expression of markers specific to chondrogenesis during chondrogenic differentiation. As a control, the differentiated PSC cells at ChD 21 2D was used. The error bars represent SD from three experiments. The statistical analysis was performed using ANOVA with Tukey’s post-hoc multicomparison test (* *p* < 0.05). ChD—Chondrogenic differentiation; 2D—two-dimensional culture; 3D—three-dimensional culture.

**Figure 3:**
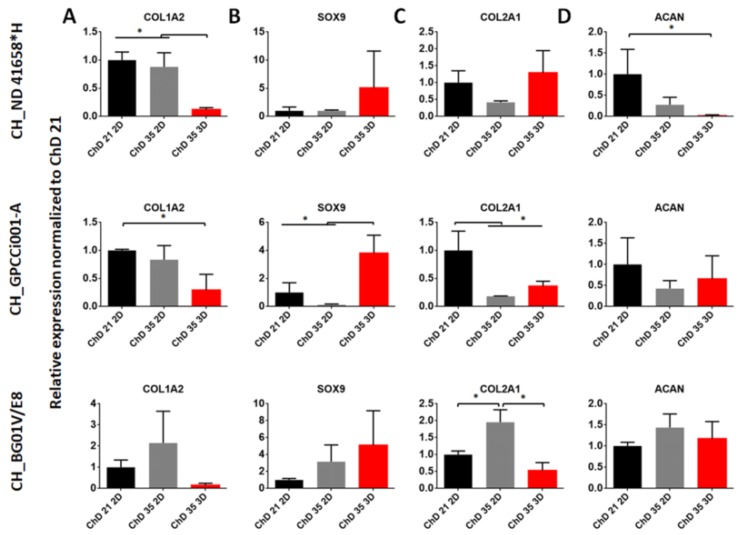
The effect of prolonged and 3D culture on the expression of chondrogenic markers. RT-qPCR represents gene expression of markers specific to chondrogenesis during chondrogenic differentiation (**A**–**D**). As a control, differentiated PSC cells at ChD 21 2D were used. The error bars represent SD from three experiments. The statistical analysis was performed using ANOVA with Tukey’s post-hoc multicomparison test (* *p* < 0.05). ChD—Chondrogenic differentiation; 2D—two-dimensional culture; 3D—three-dimensional culture.

As previously reported, we obtained the chondrogenic-like cells at the early stages of chondrogenesis, called mesenchymal condensation [29]. Thus, in this study, we decided to prolong the differentiation protocol and add a 3D culture to observe their effect on the chondrogenic differentiation of PSC in controllable conditions. For the observation of those changes, we used the RT-qPCR analysis of gene expression related to pluripotency (*NANOG*) chondrogenesis (*FGFR3, SMAD3, CDH2, NCAM1*) and chondrogenic markers (*COL1A2, SOX9, COL2A1, ACAN*) (Figure 2 and Figure 3).

The expression of *NANOG* in CH_ND41658*H increased significantly in 3D culture, and after a prolonged time of culture, in comparison with ChD 21 2D and ChD 35 3D (Figure 2A). The lack of effect was observed in CH_GPCCi001-A andCH_BG01V/E8. In CH_ND 41658*H and CH_BGV01/E8 the ChD 35 3D variant indicated an increased level of *FGFR3*, but in CH_GPCCi001-A that change was not observed (Figure 2B). Another key regulator gene, responsible for the chondrogenesis process *SMAD3* expression, was the highest in CH_ND41658*H ChD 35 3D variant. Among the studied CH_GPCC001-A variants, significant changes were not observed between ChD 21 and ChD 35 as well as in CH_BG01V/E8 (Figure 2C). A similar observation to *SMAD3* trends, among all of the studied variants of differentiated PSC, was made during the analysis of *CDH2* expression, a gene related to mesenchymal condensation (Figure 2D). Another gene involved in that process, as well as limb development, *NCAM1*, was expressed highly in CH_ND41658*H and CH_GPCCi001-A at ChD 35 in pellet culture, indicating an enhancing effect of mesenchymal condensation (Figure 2E). However, CH_BG01V/E8 exhibited a lack of significant changes between ChD 21 and ChD 35 in the used culture systems. 

Further analysis was focused on the genes related to chondrocytes. One of the genes responsible for the dedifferentiation process is *COL1A2* (Figure 3A). Its expression in all of the studied iPSC variants was reduced, but the lowest change was observed at the ChD 35 3D variant. In BG01V/E8 was no significant changes notified. Meanwhile, the increasing expression of *SOX9* was detected in prolonged 3D-culture in comparison with ChD 21 2D and Ch 35 2D (Figure 3B) only in CH_GPCC001-A. Expression of *COL2A1*, the gene encoding ECM protein responsible for the mechanical properties of hyaline cartilage, was similar between variants in CH_ND41658*H (Figure 3C). In the case of CH_GPCC001-A, the decrease of its expression was observed among the studied variants at ChD 35. The comparable expression of *COL2A1* between monolayer and pellet culture was at ChD 35. In CH_BG01V/E8, the highest expression was observed in the ChD 35 2D variant. Meanwhile, the analysis of *ACAN* expression, the gene responsible for maintaining water in the cartilage tissue, in CH_ND 41658*H decrease was noticed in ChD 35 variants in comparison with ChD 21 2D (Figure 3D). However, in CH_GPCCi001-A and CH_BGV01/E8 cells, its expression was comparable with all of the studied variants. A comparison of differentiated cells in PSC and chondrocytes indicated that the obtained cells were at the early stages of chondrogenic differentiation (Figures S1 and S2)

### 2.3. Prolonged Differentiation Increases the Deposition of ECM Specific for Chondrocytes and 3D Culture Facilitates the Formation of Chondrogenic-Like Spheres

**Figure 4:**
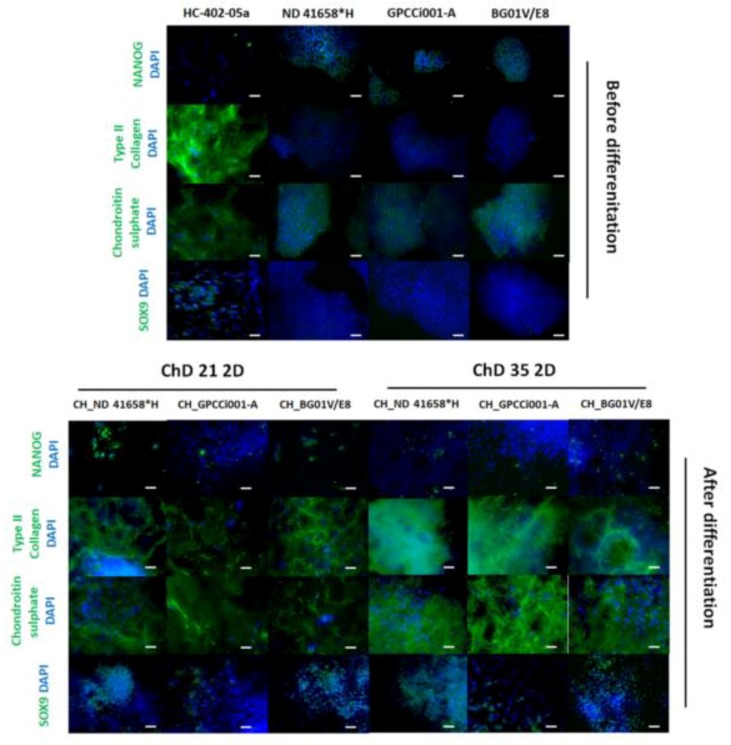
The prolonged chondrogenic differentiation in monolayer culture increased the production of ECM specific for chondrocytes. Differentiated EBs indicated a decreased number of pluripotent cells (NANOG) and increased distribution of ECM specific for chondrocytes (collagen type II, chondroitin sulfate). The white scale bars represent 100 μm. The blue correspond to stained nuclei with DAPI and the green represents protein of interest.

At ChD 35 2D culture exhibited a lack of NANOG-positively stained cells in comparison with ChD 21 2D (Figure 4 and Figure S3). Our protocol caused an increase in the deposition of the specific cartilage ECM. All of the tested differentiated PSC lines showed a stronger expression of chondrogenic markers (type II collagen, chondroitin sulfate) and more cells positively stained for SOX9. In addition, the differentiated cells represented more chondrocyte-like features than the undifferentiated pluripotent stem cells.

**Figure 5:**
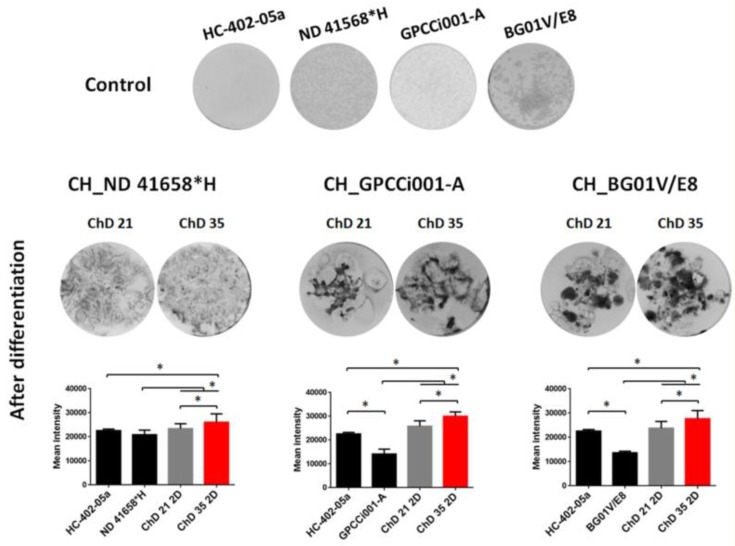
Prolonged chondrogenic differentiation in monolayer culture caused increased deposition of proteoglycans. After chondrogenic differentiation of pluripotent cell lines after 21 and 35 days of chondrogenic culture, the alcian blue staining and semi-quantification of staining density were performed. The increased deposition of proteoglycans was observed in EB outgrowth after 35 days in comparison with the 21 day and control populations. The pictures represent well of 12-well plate. The graphs represent mean density and error bars indicate SD (*n* = 6, one-way ANOVA; * *p* < 0.05).

Furthermore, to confirm the successful chondrogenic differentiation and effect of prolonged culture on ECM deposition, the alcian blue staining of 2D culture was performed and the semi-quantification of its intensity was assessed (Figure 5). In all of the differentiated PSC, the ChD 35 2D cell culture indicated the highest mean intensity in comparison with ChD 21 2D culture variants and control populations. These results also correlate with the mean intensity of chondroitin sulfate (Figure S3).

**Figure 6:**
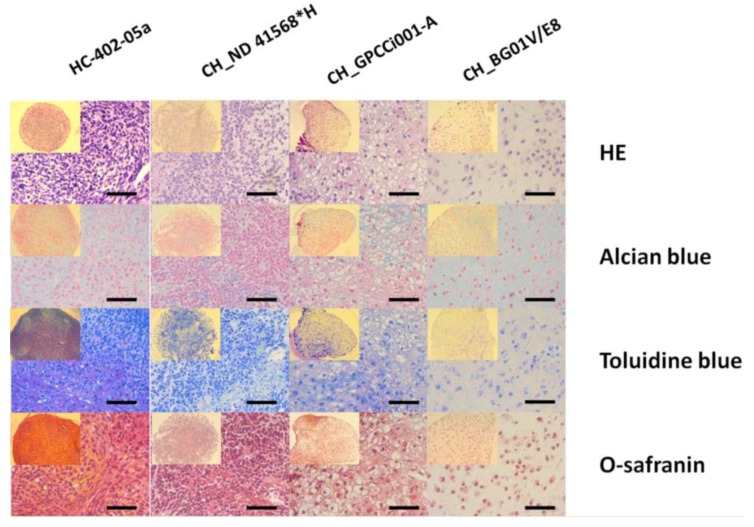
The 3D pellet culture produced premature chondrogenic like spheres. Histochemical staining for ECM has indicated the chondrocyte-like morphology of differentiated pluripotent stem cells after 35 days of chondrogenic differentiation (2 weeks in 3D culture). The positive staining for alcian blue, toluidine, and O-safranin was observed in derived chondrogenic-like spheres, but not intense like in the control population HC-402-05a cultured in 3D. The black scale bars represent 50 μm.

During in vitro culture of chondrocytes, the system of their propagation plays a major role in maintaining its biology and inhibition of the dedifferentiation process. Thus, the 3D culture after three weeks of monolayer outgrowth EBs was established. In all of the tested differentiated cells, the histochemical analysis revealed a weak positive signal for alcian blue, toluidine blue, and safranin-O in comparison with the chondrogenic pellet culture of HC-402-05a cell line (Figure 6). Moreover, in CH_BG01V/E8 the chondrogenesis process is likely to be progressing further due to the presence of formed lacunae, which is typical for the maturation of chondrocytes. These data support the RT-qPCR results (Figures S1 and S2) and the conclusion about the presence of chondrocyte-like cells in the early stages of development.

## 3. Discussion

In our study, we aimed to improve the existing protocols for chondrogenic differentiation to obtain cells at a relatively low cost of derivation, without the need to use scaffolds. Recent approaches are mostly based on direct differentiation, which calls for supplementation with an expensive combination of multiple growth factors. This leads to the formation of lateral plate mesoderm and further chondrogenic progenitors via a two- or three-dimensional approach [21,23,30]. The alternative protocols used during differentiation in specific types of cells are based on the natural ability of PSCs to form cells from the three germ layers. Previously, we have shown that the modification of cellular mass during the formation of EB can influence their differentiation in a specified germ layer and further affect the chondrogenesis process [29,31]. This was shown in BG01V cells in the presence of feeder cells and serum components, which reduces their clinical application [29]. In the present study, we used depleted animal serum and feeder cells from the differentiation process by the application of PSC cell lines adapted to those conditions and the usage for ChM chemically defined knock-out serum replacement. PSC cell lines cultured in serum- and feeder-free conditions are a well-described source of cells appropriate for pre-clinical studies [28,32]. Besides translational and reproducible aspects, the depletion of FBS during chondrogenic differentiation or chondrocytes culture has also a biological reason. It was shown that the presence of distinct serum affects the production of specific chondrogenic ECM, differentiation, and has an influence on their biomechanics properties. Several research groups have shown that during the differentiation of canine and human MSC, porcine synoviocytes, FBS induced a lower expression of type II collagen and production of glycosaminoglycans (GAGs) in comparison with serum-depleted cultures [33,34,35,36]. The reduction of serum could also decrease the presence of PSC after differentiation due to starvation, that is making the population more homogenous [21,30]. In our approach, the application of the 3D pellet culture, after three weeks of EB monolayer culture could provide the derivation of a scaffoldless chondrogenic product without the necessity of multiple passaging. This can obtain large amounts of tissue for regeneration purposes, which could reduce the occurrence of the dedifferentiation process [37,38]. The combination of these conditions mimics the avascular nature of cartilage, where nutrients are less available, and the exchange of metabolites is ensured by diffusion but not with a direct exchange with serum [39]. In our study, after the differentiation process, we observed a decreased expression of *NANOG*. However, in the BG01V/E8 cell line, mRNA was still relatively high. Immunofluorescence staining confirmed the presence of cells, positively stained for NANOG, but after a prolonged time of differentiation, their amount decreased. A similar observation was noticed in several iPSC cell-based protocols [24,30,40,41]. Further, we investigated how those conditions and proposed changes will influence the chondrogenic differentiation process via the analysis of the key role gene regulators of chondrogenesis such as *FGFR3, SMAD3, NCAM1*, and *CDH2*. *FGFR3* is essential for the regulation of endochondral ossification, and its presence regulates differentiation and proliferation of chondrogenic progenitors in the proliferation zone [22,42]. In our experiment, we assessed the increase of *FGFR3* in 3D culture, which could be related to enhanced chondrogenic processes. This was confirmed by the expression of *SMAD3*, whose expression prevents the terminal differentiation of chondrocytes and is associated with *FGFR3* during limb formation [43]. Moreover, SMAD3 is one of the regulators of COL2A1, SOX9, and aggrecan expression, narrowly regulated by the TGF-β member family [44,45]. *FGFR3* and *SMAD3* expression confirm that the 3D culture enhanced the chondrogenic response of cells mostly in the iPSC culture. However, the occurrence of the chondrogenesis process was also supported by the increased expression of genes related to mesenchymal condensation. In our differentiated cells (CH_ND 41658*H; CH_GPCCi001-A) the elevated level of the *NCAM1* transcript indicated the occurrence of mesenchymal condensation. With a parallel decreased expression of *CDH2*, which is related to the completion of the condensation process, those revelations confirm the beneficial effect of the application of the 3D microenvironment for chondrogenic differentiation [46,47]. We confirmed that our protocol leads to obtaining cells in the early stages of chondrogenesis, which was supported by the expression of chondrogenic markers. *COL1A2* expression was relatively low and *COL2A1* was increased which could indicate the presence of hyaline-like cartilage formation instead of fibrocartilage, which is typical for joint development during osteochondral ossification [48]. We also observed the deposition of specific ECM in differentiated cells, especially at ChD 35 2D. This result suggests that the promotion of chondrogenic differentiation depends on the prolongation of culture in serum-free ChM. The 3D pellets of CH_BG01V/E8 and CH_GPCCi001-A also presented the deposition of proteoglycans and the beginning of the formation of lacuna, characteristic for chondroprogenitors undergoing maturation. Similar to our protocol, Yang’s group used an embryoid body approach for hESC differentiation (H1 cell line) using KSR for all of the time frames of the differentiation process. As a result, the cartilage-like pellets were derived after 32 days of differentiation exhibiting the low intensity of toluidine blue, alcian blue, safranin-O, and type II collagen. Moreover, after eight weeks of subcutaneous sphere transplantation in a mouse model, it enabled maturation in non-hypertrophic cartilage. The obtained cells exhibited a strong intensity of a staining characteristic for cartilage [49]. A similar observation was made by Yamashita’s group. They derived hyaline cartilage-like particles after 42 days of differentiation in low-serum conditions (1% FBS). The histological analysis revealed the presence of spheres with a low deposition of ECM. However, their subcutaneous and joint implantation in the SCID rat model, enabled their maturation and the restoration of the depleted surface of joints, as well as the formation of cartilaginous tissue [50]. To sum up, the subcutaneous and joint injection of chondrocytes at the early stages of chondrogenesis is crucial for their final maturation and allows them to gain physical properties of hyaline cartilage due to microenvironmental/mechanical stimuli. In our study, a relatively low chondrogenic induction could be related to the low percentage of KSR (5%) and shorter period of differentiation in 3D culture. The recent study of Nam’s group reprogrammed cord-blood derived cells in iPSC and 47 days (30 days in 3D culture) and 20% KSR presence in the medium were sufficient for the induction of well-formed cartilage-like spheres [24,51,52]. It is worth mentioning that in their approach the induction of MSC from PSC cells was performed via the 20% FBS supplementation of monolayer culture of EB outgrowth. That approach provided a more homogenous population, but the use of animal-derived serum excludes its usefulness in clinical application [51]. In general, these studies emphasize the important fact that a microenvironment, prolonged differentiation in case of chondrogenic lineage is necessary for a derivation suitable product for regenerative purposes and this further supports our claim of obtaining an early chondrocyte-like cell population [49,50,51].

## 4. Materials and Methods

### 4.1. Cell Culture PSC

Human embryonic stem cell line (hESC) (BG01V, ATCC, Manassas, VA, USA) induced pluripotent stem cell lines (iPSC): ND*41658 (Coriel, Camden, NJ, USA) and GPCCi001-A established in our laboratory [28] were used in this study. BG01V/E8 and GPCCi001-A cell lines were adapted for feeder- and serum-free conditions as previously described [28], while the ND 41658*H cell line was commercially derived in those conditions. All the cell lines were cultured on Matrigel™ (Corning Inc., Corning, NY, USA) coated plates in DMEM:F12 supplemented with E8 reagent (Thermo Fisher Scientific Inc., Waltham, MA, USA) and 0.5% penicillin-streptomycin (Biowest, Nuaillé, France). The medium was changed every other day.

### 4.2. Chondrogenic Differentiation

The first step of the differentiation process was the formation of embryoid bodies (EB). The scheme of the experiment is presented in Figure 1A. The EB was obtained by the seeding of 500 cells/well on an ultra-low attachment 96-well plate under the previously described conditions with minor modifications [29]. A 1 mM EDTA was used for cell dissociation. In the second step, after 5 days, the formed EB were transferred onto Matrigel™-coated culture plates and were propagated in the presence of chondrogenic medium (ChM) containing: 5% Knock-out Serum Replacement (KSR, Thermo Fisher Scientific Inc., Waltham, MA, USA), DMEM-F12, 1% non-essential amino acids (NEAA, Merck KGaA, Darmstadt, Germany), 1% penicillin-streptomycin (Biowest, Nuaillé, France), 100 μM l-ascorbic acid, 100 µM l-proline, 100 nM dexamethasone (all provided by Sigma-Aldrich, Saint Louis, MO, USA), 1% ITS (Corning, Corning, NJ, USA ), and 10 ng/mL Transforming Growth Factor β3 (TGF-β3, Immunotools GmbH, Friesoythe, Germany). The medium was changed every 2 days. In order to test the influence of a prolonged and three-dimensional microenvironment on chondrogenic differentiation, some of the cells were left for another 2 weeks in monolayer culture, the remaining wells were treated with Accutase (Biowest, Nuaillé, France), filtered through a 70 μm syringe filter. Then 1.5 × 105 cells were seeded in conical 15 mL test tube in 2 mL of ChM. To form pellets, cells were centrifuged for 5 min at 200× *g*. The medium was changed every 2 days. For further analysis, the cells were collected at 26 (ChD 21) and 40 (ChD 35; 2D for a monolayer and 3D for a pellet culture) day of differentiation. Differentiated variants of the PSC cells were named as follows: CH_ND 41658*H, CH_GPCCi001-A, CH_BG01V/E8.

### 4.3. RT-qPCR

RNA was isolated from one well of 6-well (30 EB/well) plate of differentiated cells on the 3rd (ChD 21) and the 5th (ChD 35) week of differentiation, using MiniPrep RNA isolation kit (Zymo Research, Irvine, CA, USA). 1 μg of RNA was reverse transcribed in cDNA using iScript™ cDNA Synthesis Kit (Biorad, San Jose, CA, USA), according to the manufacturer’s instructions. From 20 μL of cDNA diluted 10 times, 2 μL was used for RT-qPCR. The reaction mix comprised Essential DNA Probes Master (Roche Technologies, Basel, Switzerland) and primers with dedicated molecular probes from the Universal Probe Library (Roche Technologies, Basel, Switzerland) (Table S1). All reactions were performed using Light Cycler 96 (Roche, Basel, Switzerland). For relative quantification, the B2M gene was used (Real Time Ready, Roche, Basel, Switzerland). Gene expression levels were normalized to the relevant level of mRNA in non-differentiated pluripotent cells and the adult articular chondrocytes cell line HC-402-05a (Cell Applications Inc., San Diego, CA, USA).

### 4.4. Histological Staining

After 35 days of differentiation, chondrogenic like-spheres were collected, washed in Phosphate Buffered Saline (PBS, Biowest, Nuaillé, France) and fixed in 10% buffered formaldehyde (POCH, Gliwice, Poland). After an incubation period of 24 h, spheres were embedded in a paraffin block. The prepared spheres were then sliced and stained for the presence of proteoglycans deposition by Alcian blue, Toluidine blue, O-safranin (all provided Sigma-Aldrich, Saint Louis, MO, USA). As a control, the 2-week 3D culture of chondrocytes (HC-402-05a cell line) in ChM was used. Images were then taken using an Axiovert A1 microscope (CarlZeiss, Oberkochen, Germany) under 400× magnification. 

### 4.5. Immunofluorescent Staining of EB Outgrowth

After 3 and 5 weeks of chondrogenic differentiation, cells were fixed in ice-cold methanol (POCH, Gliwice, Poland) and incubated at −20 °C. Later, the cells were washed and blocked for non-specific binding using 1% bovine serum albumin (BSA, AppliChem GmbH, Darmstadt, Germany) containing 0.5% Tween 20 (POCH, Gliwice, Poland) for 30 min. The cells were then incubated overnight at 4 °C with primary antibodies (Table S2). The next day, cells were washed 3 times in PBS containing 1% BSA and underwent 1h incubation with secondary antibodies at 37 °C (Table S2). Nuclei were counterstained with 4′,6-diamidino-2-phenylindole (DAPI, Sigma-Aldrich, Saint Louis, MO, USA) to show the nucleus. Images were taken using an Opta-Tech MW-100 microscope (Opta-Tech, Warsaw, Poland) under 200× magnification and documented using Opta-View software (Opta-Tech, Warsaw, Poland). The pictures were then merged and semiquantified as previously described [53] using an Image-J ver 1.50i (National Institute of Mental Health, Bethesda, MA, USA). 

### 4.6. Determination of Proteoglycans Deposition in 2D Culture

To assess the deposition of proteoglycans in the differentiated cells, alcian blue staining was performed as previously described [29]. Pictures were taken using ChemiDoc-Touch (Biorad, Hercules, CA, USA), at the same exposure time (*t* = 0.594 s). In order to quantify the proteoglycans deposition, the mean intensity signal of the picture from the whole well of 12-well plate (at least the mean from three wells) was calculated using dedicated software ImageLab (Biorad, Hercules, CA, USA) and normalized to the mean staining intensity of HC-402-05a cultured in monolayer. 

### 4.7. Statistical Analysis

The analyses were performed in at least three independent experiments. The calculations were performed using a GraphPad Prism 6 (Graph Pad Software, San Diego, CA, USA). Significant differences for *p* < 0.05 were acknowledged.

## 5. Conclusions

In our study, we differentiated PSC cells adapted to serum- and feeder-free conditions in chondrogenic-pellets at the early stage of chondrogenesis in controllable and reproducible conditions. We believe the chemically-defined conditions might enable the clinical use of our protocol.

## Supplementary Materials

Supplementary materials can be found at https://www.mdpi.com/1422-0067/20/11/2711/s1. Figure S1: The effect of prolonged and 3D culture on pluripotent, chondrogenic signaling pathways and mesenchymal condensation gene markers. Figure S2: The effect of prolonged and 3D culture on the expression of chondrogenic markers. Figure S3: The semiquantification of immunofluorescence staining of differentiated cells. Table S1: List of primers. Table S2: List of antibodies.

## Abbreviations

OA	Osteoarthritis
iPSCs	Induced Pluripotent Stem Cells
3D	Three-dimensional
ECM	Extracellular Matrix
ACI	Autologous Chondrocyte Implantation
MSC	Mesenchymal Stromal Cells
PSC	Pluripotent Stem Cells
hESC	Human Embryonic Stem Cells
FBS	Fetal Bovine Serum
SOX2	SRY (sex determining region Y)-box 2
OCT4	Octamer-binding transcription factor 4
CDH1	Cadherin-1 (E-cadherin)
TGF-β3	Transforming Growth Factor beta 3
EB	Embryoid Body
ChD	Chondrogenic Differentiation
ChM	Chondrogenic Medium
PHDF	Primary Human Dermal Fibroblast
RT-qPCR	Reverse Transcriptase quantitative Polymerase Chain Reaction
FGFR3	Fibroblast Growth Factor Receptor 3
SMAD3	SMAD Family Member 3
CDH2	Cadherin-2 (N-cadherin)
NCAM1	Neural Cell Adhesion Molecule 1
COL1A2	Collagen type 1 alpha 2 chain
SOX9	SRY (sex determining region Y)-box 9
COL2A1	Collagen type 2 alpha 1 chain
ACAN	Aggrecan
GAGs	Glycosaminoglycans
KSR	Knock-out Serum Replacement
SCID	Severe Combined Immunodeficiency
DMEM	Dulbecco Modified Eagle Medium
PBS	Phosphate Buffered Saline
EDTA	Ethylenediaminetetraacetic acid
NEAA	Non-essential Amino Acids
B2M	Beta-2-Microglobulin
DAPI	4′,6-diamidino-2-phenylindole
