# Supplementary material for: Chondrogenic Differentiation of Pluripotent Stem Cells under Controllable Serum-Free Conditions
Source: Int J Mol Sci. 2019 Jun 2;20(11):2711. doi: 10.3390/ijms20112711 (PMC6600514; doi:10.3390/ijms20112711)
Supplement: Supplementary file 1 [file ijms-20-02711-s001.pdf]

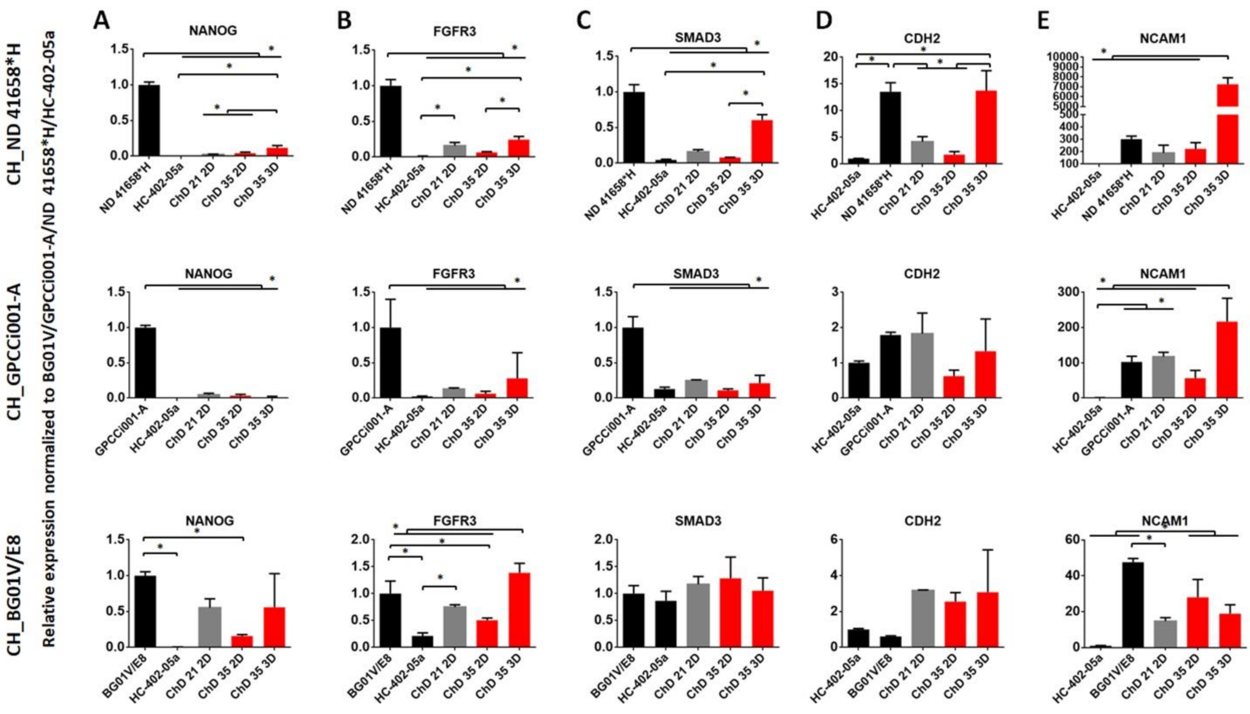

Figure S1. The effect of prolonged and 3D culture onto pluripotent (A), chondrogenic signalling pathways (B, C) and mesenchymal condensation (D, E) gene markers. RT-qPCR represents gene expression of markers specific to chondrogenesis during of chondrogenic differentiation. As a control the HC-402-05a was used and non-differentiated pluripotent stem cells. The error bars represent SD from three experiments. The statistical analysis was performed using ANOVA with Tukey's post-hoc multicomparison test (* - p<0,05). ChD – Chondrogenic differentiation; 2D – two-dimensional culture; 3D – three-dimensional culture.

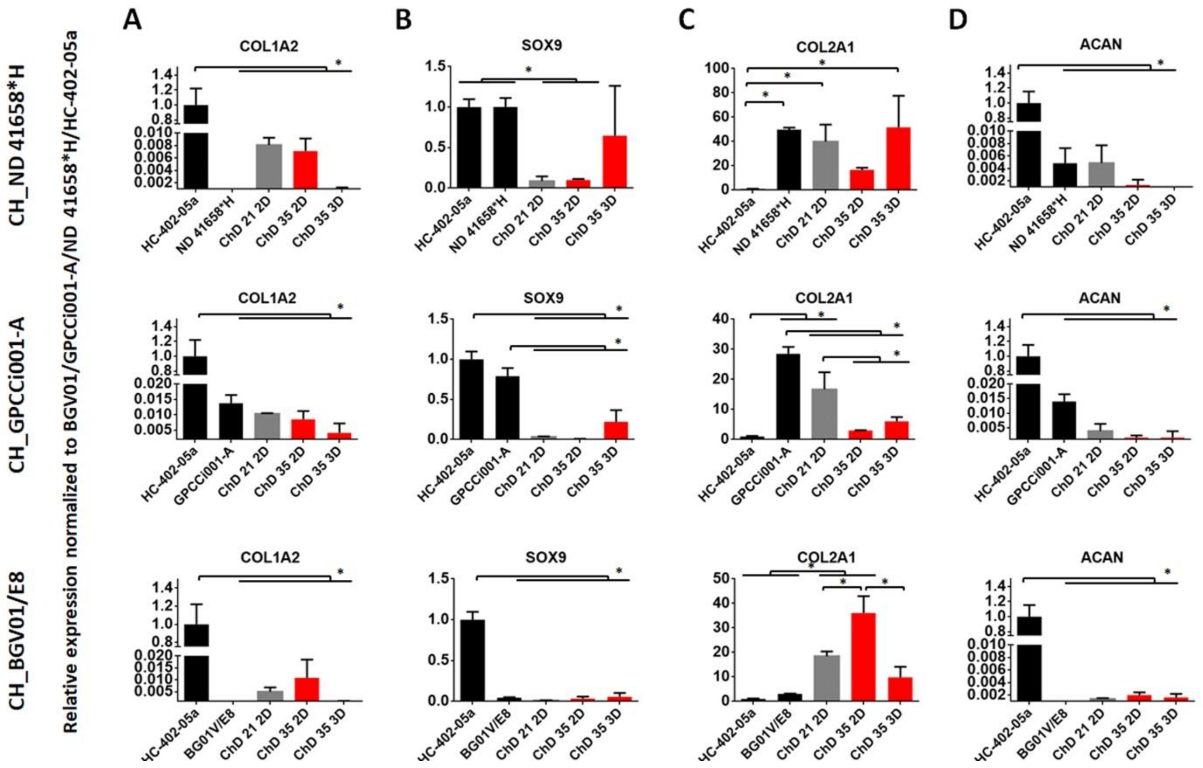

Figure S2. The effect of prolonged and 3D culture onto expression of chondrogenic markers. RT-qPCR represents gene expression of markers specific to chondrogenesis during chondrogenic differentiation (A, B, C & D). As a control the HC-402-05a was used and non-differentiated pluripotent stem cells. The error bars represent SD from three experiments. The statistical analysis was performed using ANOVA with Tukey's post-hoc multicomparison test (* - p<0,05). ChD – Chondrogenic differentiation; 2D – two dimensional culture; 3D – three-dimensional culture.

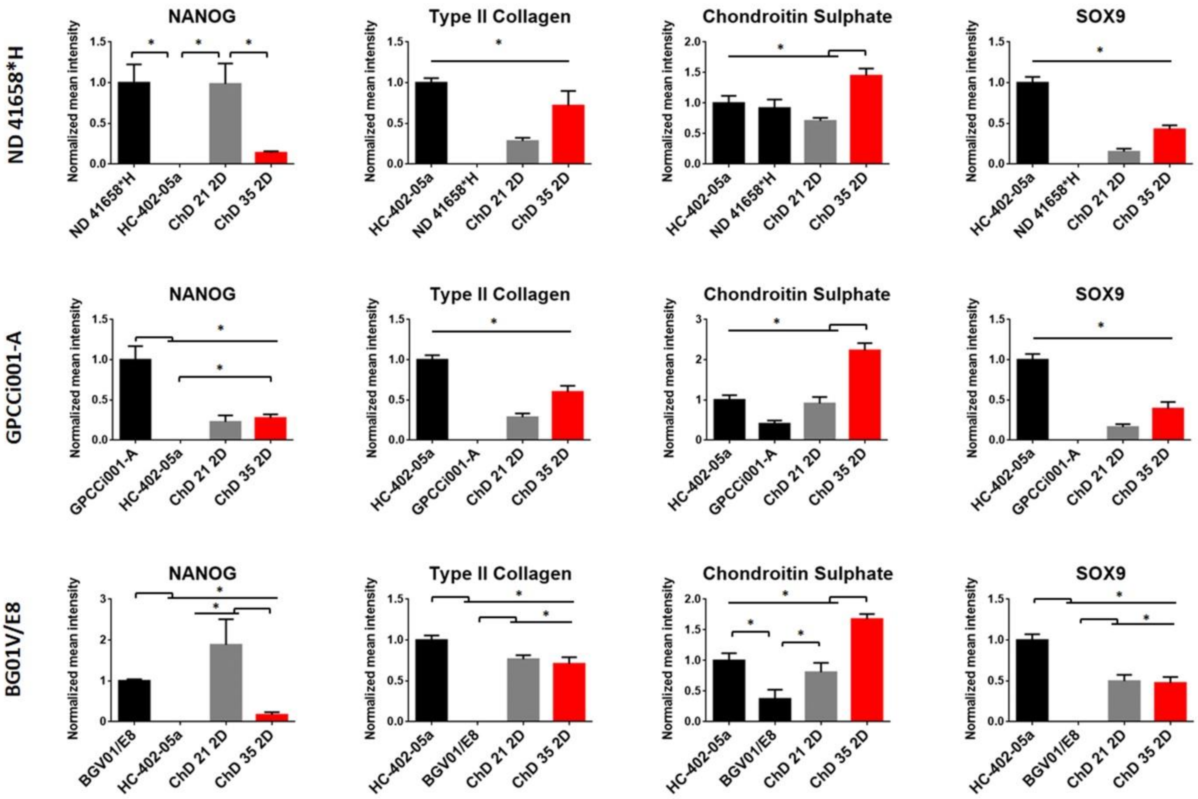

Figure S3. The semi-quantification of immunofluorescence staining for pluripotent and chondrogenic markers of differentiated and controls cell populations. Decreased mean intensity of NANOG and type II collagen and SOX9 of differentiated PSC populations was observed in comparison with controls. The chondroitin sulphate intensity was the highest at ChD 35 of PSC chondrogenic differentiation. As a control the HC-402-05a was used and non-differentiated pluripotent stem cells. The error bars represents SD from three experiments. The statistical analysis was performed using ANOVA with Tukey's post-hoc multicomparison test (* - p<0,05). ChD – Chondrogenic differentiation; 2D – two dimensional culture; 3D – three-dimensional culture.

Table S1. List of primers

Target mRNA	Forward Sequence	Reverse Sequence	Reference sequence	Probe no.
ACAN	cgtgaatcagaatcaactgctg	gtgtccctctgtctccttgc	*NM_013227.3*	*31*
CDH1	aagttttccaccaaagtcacg	tgcttggattccagaaacg	*NM_004360.3*	*77*
CDH2	atccgacgaatggtgaaag	ctgtggggtcattgtcagc	*NM_001792.3*	*45*
COL1A2	ggcagtgatggaagtgtgg	ccaacagctccaatttcacc	*NM_000089.3*	*45*
COL2A1	ttctggagaccaaggtgctt	ttccattagcaccatctttgc	*NM_001844.4*	*67*
FGFR3	agaggcccaccttcaagc	cgacaggtccaggtactcgt	*NM_000142.4*	*22*

NANOG	atgcctcacacggagactgt	aagtgggttgtttgcctttg	*NM_024865.2*	*31*
NCAM1	cgaccatccacctcaaagtc	cggaggcttcacaggtaaga	*NM_000615.6*	*68*
OCT4	cttcggatttcgccttctc	cttagccaggtccgaggat	*NM_002701.4*	*77*
SMAD3	caccacgcagaacgtcaa	gatgggacacctgcaacc	*NM_005902.3*	*9*
SOX2	tgcctctttaagactaggactgaga	gccgccgatgattgttatta	*NM_003106.3*	*45*
SOX9	ctcgccacactcctcctc	cgcttcaggtcagccttg	*NM_000346.3*	*77*

Aggrecan ACAN; E-cadherin – CDH1; N-cadherin – CDH2; Type 1 collagen - COL1A2; Type II collagen - COL2A1; Fibroblast Growth Factor Receptor 3 - FGFR3; Neural Cell Adhesion Molecule 1 - NCAM1; Octamer-binding transcription factor 4 – OCT4; SMAD Family Member 3 - SMAD3; SRY (sex determining region Y)-box 2 – SOX2; SRY (sex determining region Y)-box 9 - SOX9.

Table S2. List of antibodies

Primary antibodies	Host / Isotype	Vendor	Dilution
**NANOG**	Mouse / IgG1	BD Pharmigen, CA, USA	1:50
**SOX9**	Rabbit / IgG	Merck-Millipore, Darmstadt Germany	1:125
**Type II collagen**	Rabbit / IgG	Abcam, Cambridge, UK	1:100
**Chondroitin Sulphate**	Mouse / IgM	Abcam, Cambridge, UK	1:100
**Secondary antibodies**	**Host / Isotype**	**Vendor**	**Dilution**
**Anti-Mouse IgG conjugated with Alexa Fluor 488**	Donkey/ IgG	Jackson ImmunoResearch, PA, USA	1:500
**Anti-Rabbit IgG conjugated with Alexa Fluor 488**	Donkey/ IgG	Jackson ImmunoResearch, PA, USA	1:500
**Anti-Mouse IgM conjugated with Alexa Fluor 488**	Donkey/ IgG	Jackson ImmunoResearch, PA, USA	1:500
